# Real-time chlorophyll fluorescence monitoring reveals dynamic acclimation of lettuce to temperature and light stress in controlled environments

**DOI:** 10.3389/fpls.2026.1733839

**Published:** 2026-02-10

**Authors:** Suyun Nam, Rhuanito Soranz Ferrarezi

**Affiliations:** Department of Horticulture, University of Georgia, Athens, GA, United States

**Keywords:** abiotic stress, chlorophyll fluorescence, controlled environment agriculture, light acclimation, photosynthetic efficiency, temperature stress

## Abstract

Real-time monitoring of photosynthetic efficiency can improve our understanding of plant stress responses. In this study, we used a high-frequency chlorophyll fluorescence (CF) monitoring system to investigate the effects of combined temperature and light effects on lettuce. Plants were exposed to three temperatures (18, 25, and 32 °C) and two light intensities (150 and 500 μmol·m^-2^·s^-1^) for one week, and CF parameters were measured every 30 minutes. Gas exchange measurements were conducted at 2 and 7 days after treatment (DAT). High light combined with low temperature initially suppressed Φ_PSII_ but gradually improved via reductions in quantum yield of non-regulated energy dissipation (Φ_NO_), indicating adjustments in the photosynthetic machinery. While the quantum yield of non-photochemical quenching (Φ_NPQ_) decreased sharply only on the first day, Φ_NO_ continued to decline, highlighting its role in longer-term acclimation. In contrast, high temperatures enhanced CO_2_ assimilation through elevated stomatal conductance; however, the maximum efficiency of PSII (*F*_v_/*F*_m_) remained suppressed (~0.81), suggesting sustained photoinhibition. The relationship between electron transport rate (ETR) and photosynthetic rate (*A*) varied with temperature and time, indicating that the efficiency of converting photochemical energy into carbon assimilation depended on stress conditions and the acclimation stage. However, cumulative ETR integrated over the experiment period was significantly associated with shoot dry weight independent of temperature conditions, indicating that temporally integrated CF metrics retain predictive value for growth, unlike instantaneous CF parameters. These findings demonstrate that high-resolution CF monitoring captures subtle and dynamic photosynthetic responses that are not detectable via single-point gas exchange measurements alone. The ability to interpret changes in CF parameters in real-time provides valuable insights into plant acclimation and stress physiology for the optimization of environmental conditions in controlled environment agriculture systems.

## 1 Introduction

Maintaining optimal photosynthetic performance in controlled environment agriculture (CEA) facilities is challenging under overcast or substandard environmental conditions. Limited or excessive light and temperature can induce physiological stress and increase reliance on energy-intensive environmental control, including supplemental lighting, shading systems, and heating, ventilation, and air conditioning (HVAC) systems. Therefore, optimizing photosynthetic efficiency is critical for maximizing crop productivity and improving energy use efficiency in CEA. High light intensity and temperature stress are among the most common environmental constraints in CEA production systems (Zhou et al., 2022). In general, photosynthetic efficiency decreases under excessive light, because surplus absorbed light energy is dissipated as heat and chlorophyll fluorescence to prevent photodamage to photosystem II (PSII) (Wimalasekera, 2019). Both heat and cold stress reduce photosynthetic efficiency and impair plant growth, primarily through the generation of reactive oxygen species (ROS) and photodamage to PSII components (Hussain, 2019).

However, the effect of high light and temperature stress varies depending on the combination of stressors and the duration of exposure. In particular, low temperatures combined with high light intensities can synergistically induce severe photoinhibition due to the overexcitation of PSII reaction centers (Janda et al., 2021). Prolonged exposure to both high light and high temperatures may lead to the irreversible inactivation of PSII due to the downregulation of PSII protein gene expression and associated repair mechanisms (Lu et al., 2017). Although this combination of stressors can mitigate oxidative damage through the accumulation of carbohydrates and carotenoids as protective and adaptive strategies, its effectiveness depends on the severity of the stresses and the crop species (Zhou et al., 2020).

Moreover, the stress responses are crop-specific and evolve over time, influenced by “stress memory”, defined as a history of exposure to different types of environmental stresses (Bruce et al., 2007; Yamori et al., 2014). For instance, the photosynthetic efficiency of lettuce (*Lactuca sativa*) increased significantly within a single day under high light, whereas cucumber (*Cucumis sativus*) maintained a constant level, indicating a species-specific response (Nam et al., 2025). Over longer periods, plants exhibit dynamic acclimation to light and temperature stresses through diverse physiological mechanisms such as chloroplast relocation, accumulation of accessory pigments, membrane fluidity adjustments, and leaf morphological changes (Wimalasekera, 2019). Monitoring real-time plant stress responses is essential for understanding how environmental factors dynamically influence photosynthetic performance. While single-leaf gas exchange remains a conventional method for assessing photosynthetic capacity and stomatal behavior, it is time-consuming and not suitable for high-frequency or long-term real-time monitoring. Consequently, temporal dynamics and acclimation patterns are often overlooked (Haworth et al., 2023). Whole-plant gas exchange systems enable the continuous measurement of whole-canopy photosynthesis, but their complexity reduces their practical applicability (Sakoda et al., 2025; van Iersel and Bugbee, 2000). Other traditional physiological assessments, such as biochemical or pigment analyses, are labor-intensive and destructive. Even though these more sophisticated systems have limited use, high-temporal-resolution monitoring is therefore critical for early stress detection, capturing within-day and long-term variability, and improving our understanding of plant-environment interactions (Haworth et al., 2023).

Chlorophyll fluorescence (CF) monitoring, based on the pulse amplitude modulation (PAM) technique, is a rapid, non-destructive, and highly sensitive method for detecting environmental stress. Parameters such as the maximum efficiency of PSII (*F*_v_/*F*_m_) serve as diagnostic indicators of photoinhibition under extreme temperature or excess light (Lysenko et al., 2022). Because CF measurements are non-destructive, repeated sampling enables detection of diurnal changes and acclimation responses. Linear electron transport rate (ETR) is generally well correlated with carbon dioxide (CO_2_) assimilation, offering a potential proxy for crop productivity. Additionally, CF allows detailed analysis of dynamic photochemical quenching, including regulated and unregulated energy dissipation (Lysenko et al., 2022; Maxwell and Johnson, 2000).

CF has been applied for early abiotic stress detection, crop phenotyping, prediction of flowering time, and development of productivity models (Hussain, 2019; Kalaji et al., 2016; Yu and Chen, 2023). It has also been implemented in real-time environmental control systems for artificial lighting. For example, a CF-based biofeedback system can collect CF parameters every 15 minutes via serial communication with a datalogger to dynamically adjust the lighting-emitting diode (LED) light intensity (Nam et al., 2025; van Iersel et al., 2016).

However, under photorespiratory stress conditions such as heat or drought, the relationship between ETR and carbon assimilation may weaken due to low CO_2_ availability (Lysenko et al., 2022). This can cause a decoupling between fluorescence parameters and the actual carbon assimilation, which may limit the reliability of CF as a proxy for photosynthetic performance under certain stressful conditions. This response should be better studied to understand the factors needed to control lighting successfully.

The overall objective of this study was to demonstrate the utility of a high-frequency CF monitoring system for assessing plant physiological responses under temperature and light stress over time. It was hypothesized that high-frequency CF measurements can resolve temperature- and light-dependent stress responses and their interaction and acclimation dynamics over time, and that CF-derived metrics are associated with downstream physiological outcomes, including carbon assimilation and crop growth under stress conditions. This knowledge can be used for further automated lighting control using CF as a biofeedback control system.

## 2 Materials and methods

### 2.1 Location

The study was conducted at the University of Georgia (College of Agricultural and Environmental Sciences, Department of Horticulture, Controlled Environment Agriculture Crop Physiology and Production Laboratory) in Athens, Georgia, USA (33 93’11.36” N, 83 36’39.28” W).

### 2.2 Plant material, growth conditions

Lettuce ‘Green Towers’ seeds (Johnny’s Selected Seeds, Waterville, ME, USA) were sown into 10-cm square containers filled with a peat-perlite soilless substrate (Fafard 1P; SunGro Horticulture, Agawam, MA, USA). Seedlings were grown in a walk-in growth chamber illuminated with white LED light bars (RAY series with Physiospec indoor spectrum; Fluence Bioengineering, Austin, TX, USA) emitting 39% red, 40% green, 18% blue, and 3% far-red. Canopy-level photosynthetic photon flux density (PPFD) was maintained at 250 μmol·m^-2^·s^-1^, with a 16-h photoperiod (daily light integral [DLI] of 14.4 mol·m^-2^·d^-1^). Irrigation was supplied daily by an automated ebb-and-flow subirrigation system using a 15N-2.2P-12.4K fertilizer (Jack’s Professional^®^ LX 15-5–15 Cal-Mg LX; JR Peters, Allentown, PA, USA) at 100 mg·L^-1^ N. The average environmental conditions were a temperature of 23.7 ± 0.2 °C, vapor pressure deficit (VPD) of 1.5 ± 0.2 kPa, and CO_2_ concentration of 814.2 ± 28.7 μmol·mol^-1^ (mean ± standard deviation).

At 3 weeks after seeding, the plants with 6–8 true leaves were transferred to an experimental growth chamber (E15; Conviron, Winnipeg, Manitoba, Canada) under ambient CO_2_ and hand-watered daily with the same nutrient solution. The chamber was equipped with six 200-W white LED bars (Rev-2: GrowRay, Boulder, CO, USA) with 50% red, 20% green, 24% blue, and 6% far-red, mounted 40 cm above the canopy. Spectral distributions were verified using a spectrometer (LI-180; LI-COR Biosciences, Lincoln, NE, USA). The lighting operated from 0:00 to 16:00 (16 h).

### 2.3 CF measurement

CF was measured on the same uppermost fully expanded leaf throughout the 7-day period to track temporal responses to treatments. A pulse-amplitude modulated (PAM) fluorometer (MINI-PAM; Heinz Walz, Effeltrich, Germany), remotely controlled by a datalogger (CR1000; Campbell Scientific, Logan, UT, USA) via an RS-232 interface, was used for *in situ* measurements in the growth chamber. Through serial communication, the datalogger automatically executed measurement commands, retrieved data, and calculated the fluorescence parameters (Nam et al., 2025).

Every 30 minutes during the 16-h photoperiod, saturating light pulses were applied to determine maximum fluorescence in light (*F*_m_′), with steady-state fluorescence (*F*_t_) measured immediately beforehand. The white LED light was then briefly turned off, and a 2-second pulse of far-red light was applied to measure minimal fluorescence in light (*F*_o_′), after which white light resumed. The operating PSII efficiency (Φ_PSII_) was calculated as (*F*_m_′ – *F*_t_)/*F*_m_′ (Genty et al., 1989) to estimate the proportion of absorbed light used in photochemistry. ETR, representing the overall photosynthetic capacity, was estimated as Φ_PSII_ × PPFD × 0.5 × 0.84, assuming an equal distribution of photons between PSI and PSII and an 84% incident light absorption (Maxwell and Johnson, 2000).

Light-adapted maximum quantum efficiency (*F*_v_′/*F*_m_′) was calculated as (*F*_m_′ – *F*_o_′)/*F*_m_′, which represents the maximum efficiency of PSII while acclimated at a given light intensity (Baker et al., 2007). Dark-adapted parameters (*F*_o_ and *F*_m_) were measured hourly from 16:00 to 0:00, and dark-adapted maximum efficiency of PSII (*F*_v_/*F*_m_) was calculated as (*F*_m_ – *F*_o_)/*F*_m_. *F*_v_/*F*_m_ measured after 1 and 8 hours of dark adaptation (*F*_v_/*F*_m_ 1h and *F*_v_/*F*_m_ 8h) were used to assess PSII recovery, as photoprotective and photoinhibitory components of non-photochemical quenching (NPQ) relax at different rates, ranging from minutes to several hours, depending on stress severity (Maxwell and Johnson, 2000). Lake model-based photochemical quenching coefficient (qL) represents the fraction of PSII reaction centers that are open. Quantum yield of NPQ (Φ_NPQ_) and quantum yield of non-regulated energy dissipation (Φ_NO_) were calculated following Kramer et al. (2004).


Photochemical quenching coefficient (qP)=(Fm'–Ft)/(Fm'–Fo')

qL=qP×(Fo'/Ft)

NPQ=(Fm–Fm')/Fm'

$${\text{Φ}}_{\text{NO}}=1/(\text{NPQ}+1+\text{qL}\times ((F_{\text{m}}/F_{\text{o}})–1))$$

$${\text{Φ}}_{\text{NPQ}}=1–{\text{Φ}}_{\text{PSII}}–{\text{Φ}}_{\text{NO}}$$

Measurements were taken automatically by the datalogger using a proprietary software (LoggerNet v.4.7; Campbell Scientific, Logan, UT, USA). The datalogger triggered measurements of *F*_m_/*F*_m_′, *F*_o_/*F*_o_′, and *F*_t_ every 30 minutes during the photoperiod and hourly during the dark period. These raw fluorescence values were subsequently used to calculate various CF parameters. Additionally, with the integration of far-red LED light into the automated CF measurement protocol, parameters that require *F*_o_′ to be calculated, such as *F*_v_′/*F*_m_′ and qL, can be obtained throughout the daytime in the presence of ambient light. Cumulative ETR was calculated by integrating instantaneous ETR values measured at 15-min intervals during the photoperiod across the entire experimental period. This metric quantifies the total photochemical electron flux processed by PSII over time and was used to evaluate the relationship between integrated photochemical activity and final shoot biomass.

### 2.4 Leaf photosynthesis measurements and harvest

Leaf gas exchange was measured on the uppermost fully expanded leaves at two days after treatment (DAT) and DAT 7 using a portable photosynthesis system (CIRAS-3; PP Systems, Amesbury, MA, USA). Net photosynthetic rate (*A*), stomatal conductance (*g*_s_), transpiration rate (*E*), intercellular CO_2_ concentration (*C*_i_), and water use efficiency (WUE) were recorded under a constant CO_2_ concentration of 400 μmol·mol^-1^, reflecting typical ambient CO_2_ levels for standardized measurements.

At DAT 7, rapid *A*–*C*_i_ response curves were obtained using a high-speed CO_2_ ramping technique (Stinziano et al., 2017) to assess photosynthetic capacity and identify limiting factors under experimental stress conditions. CO_2_ concentration was ramped from 100 to 1500 μmol·mol^-1^ over 6 minutes, with baseline cuvette response established using an empty cuvette. Post-data processing was conducted to recompute actual *A* and *C*_i_ values using a spreadsheet provided by the portable photosynthesis system. Curve fitting was then performed by minimizing the residual sum of squares, as described by Sharkey et al. (2007), to derive the maximum rate of ribulose-1,5-bisphosphate carboxylase oxygenase (Rubisco) carboxylation (*V*_c,max_), the maximal rate of electron transport (*J*_max_), and triose phosphate utilization (TPU). More details regarding the methodology of the rapid *A*/*C*_i_ curves can be found in PPSystems (2022) and Liu and van Iersel (2021).

While gas exchange parameters at DAT 2 were directly measured under a steady CO_2_ concentration, those at DAT 7 were extracted from the ramping data point at 400 μmol·mol^-1^ CO_2_. During all gas exchange measurements, environmental conditions in the cuvette (temperature, light intensity, and humidity) were set to match the corresponding experimental conditions. Measurements were conducted between 14:00 and 16:00 to minimize the effect of the time of day.

All lettuce plants were harvested at DAT 7. Shoot fresh weight was measured immediately, and shoot dry weight was determined after oven-drying at 80°C for 72 hours. Shoot water content was measured to evaluate stress-induced dehydration and morphological adaptation, and it was calculated as (shoot fresh weight – shoot dry weight)/shoot fresh weight. Leaf chlorophyll content was measured using a handheld chlorophyll meter (CCM-220 plus; Opti-Sciences, Hudson, NH, USA) on six uppermost fully expanded leaves per plant, and the values were averaged.

### 2.5 Treatments and experimental design

Plants were exposed to three temperatures (18, 25, and 32°C) and two light intensities (150 and 500 µmol·m^-2^·s^-1^ PPFD) for 7 days. The temperature treatments were selected to span a physiologically relevant range for lettuce, with 25 °C representing near-optimal daytime conditions and 18 and 32 °C representing cooler and warmer conditions that may induce stress (Holmes et al., 2019; Zhou et al., 2022). The two PPFD levels, applied under a 16-h photoperiod, corresponded to DLI of 8.64 and 28.8 mol·m^-2^·d^-1^, representing typical greenhouse light conditions, within ranges where lettuce growth increases with PPFD and DLI (Faust and Logan, 2018; Mayorga-Gomez et al., 2024).

The actual average temperatures and light levels recorded across all replicates during the treatment period were 18.1 ± 1.0, 24.8 ± 0.3, and 31.9 ± 0.4 °C, and 150.0 ± 0.6 and 500.7 ± 2.7 µmol·m^-2^·s^-1^, respectively. Light intensity was continuously monitored using photodiodes (SLD-69C1; Silonex, Montreal, Quebec, Canada), with one sensor placed at canopy height per experimental unit. All photodiodes were calibrated against a quantum sensor (MQ-500; Apogee Instruments, Logan, UT, USA) before the experiment and connected to a datalogger for real-time monitoring and light adjustment.

The average VPD was 0.99 ± 0.16, 1.20 ± 0.18, and 1.21 ± 0.22 kPa, and the corresponding relative humidity (RH) values were 52.7% ± 5.4%, 61.6% ± 5.7%, and 74.5% ± 4.4% at 18, 25, and 32°C, respectively. In the growth chamber, humidity was controlled to balance VPD among temperature treatments. At 32°C, humidifiers (Classic 300S Ultrasonic Smart Humidifier; Levoit, Anaheim, CA, USA) were used to maintain approximately 75% RH, whereas at 18°C, two dehumidifiers (800 sq ft dehumidifier; Gocheer, Shenzhen, China) operated at full capacity to reduce RH and increase VPD, although values above ~ 1.2 kPa could not be achieved.

The experiment was arranged as a split-plot randomized complete block design (RCBD) conducted over 12 weeks in a single growth chamber. Due to spatial limitations, blocks were arranged temporally, with each 3-week period considered one block and the sequence repeated four times, resulting in a total of 12 weeks. Temperature (whole-plot factor) was randomly assigned weekly to each experimental unit (7-day period) within each block (n = 4). A reflective partition divided the chamber into two sections (left and right) for the light treatments (split-plot factor), with 150 and 500 µmol·m^-2^·s^-1^ PPFD applied at each light intensity randomly within each temperature condition. The temperature was uniform across the chamber during the experimental period.

### 2.6 Statistical analysis

All statistical analyses were conducted in statistical software (R version 4.5.0; R Foundation for Statistical Computing, Vienna, Austria). Linear mixed-effects models were used for all analyses, with experimental block included as a random effect. For CF parameters, three-way analysis of variance (ANOVA) was performed with temperature, PPFD, and DAT as fixed factors. The fixed-effects structure of the model was y_ijkl_ = µ + T_i_ + P_j_ + D_k_ + (TP)_ij_ + (TD)_ik_ + (PD)_jk_ + (TPD)_ijk_ + b_l_ + ϵ_ijkl_, where T, P, and D represent the effects of temperature, PPFD, and DAT effect, respectively, b_l_ represents the random effect of experimental block, and ϵ_ijkl_ is the residual error. Because CF parameters were measured repeatedly over time, a continuous autoregressive correlation structure (corCAR1) was used to account for temporal autocorrelation among observations. Variance weighting was also applied to address heteroscedasticity observed across temperature and PPFD treatments. Model structures were evaluated sequentially, and autocorrelation functions (ACF) of normalized residuals were used to confirm that temporal autocorrelation was adequately accounted for without introducing unnecessary model complexity. For gas exchange, *A*/*C*_i_ curve, and harvest parameters, two-way ANOVA was conducted with temperature and PPFD as fixed factors. Pairwise comparisons were performed using Tukey’s Honestly Significant Difference (HSD) test at a 95% confidence level. For the correlation between ETR and *A*, linear models were constructed that included interaction terms of ETR with temperature, DAT, and the interaction of temperature × DAT to test how these factors influenced the slope of the *A*–ETR relationship. Model assumptions were evaluated using residual diagnosis, including residual-fitted plots and quantile-quantile plots, to confirm homoscedasticity and normality of residuals.

## 3 Results

### 3.1 Real-time monitoring of photochemical activities

**Figure 1:**
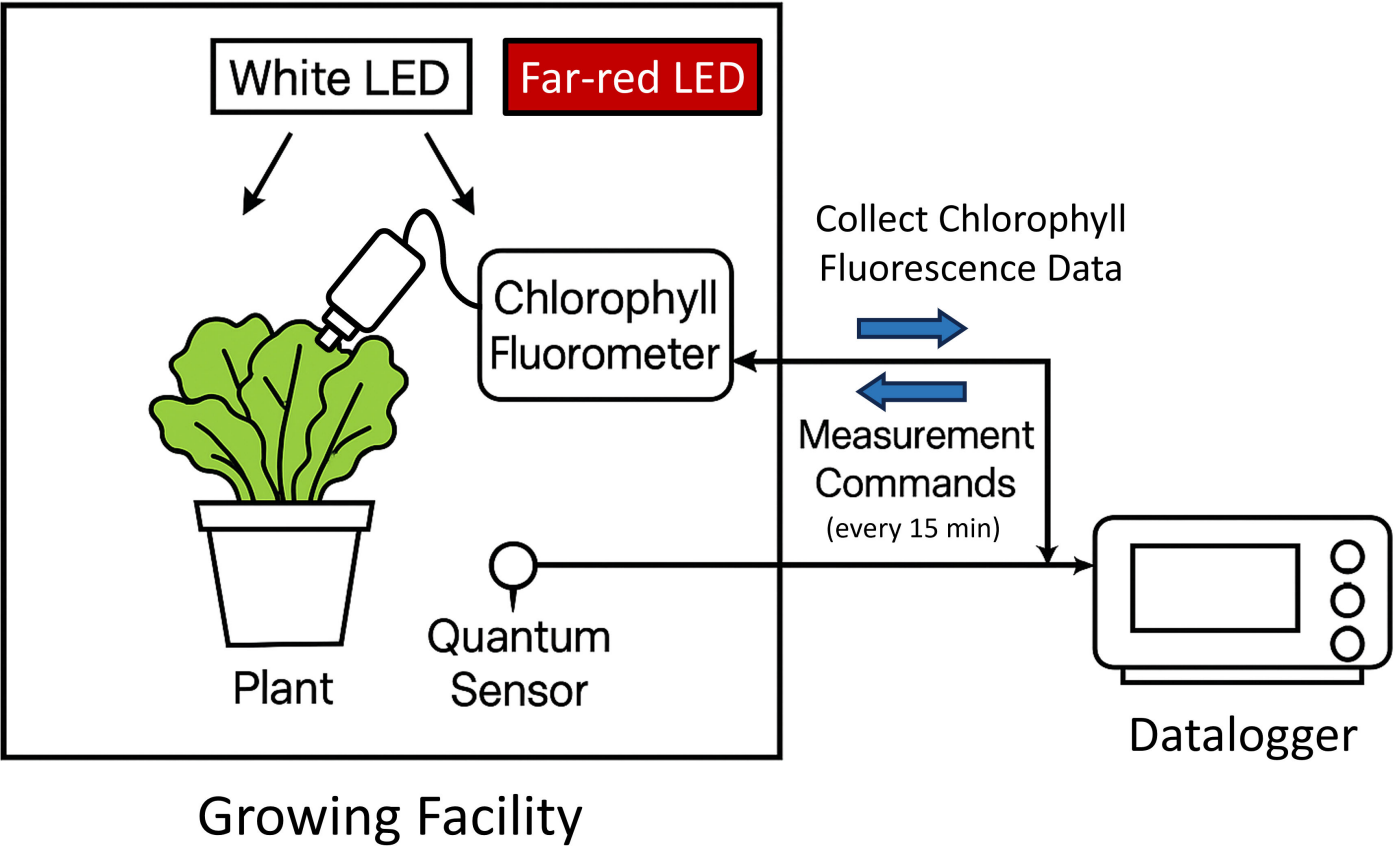
Schematic of the real-time chlorophyll fluorescence (CF) monitoring system installed in a growth chamber. The data logger communicates with the chlorophyll fluorometer via serial communication to send measurement commands at specified intervals and receive real-time CF data. White light emitting diodes (LEDs) provide actinic light for plant growth. During each CF measurement cycle, the white LEDs are temporarily turned off, and far-red LEDs activate for a few seconds to allow measurement of minimal fluorescence. After the measurement, actinic lighting resumes.

**Figure 2:**
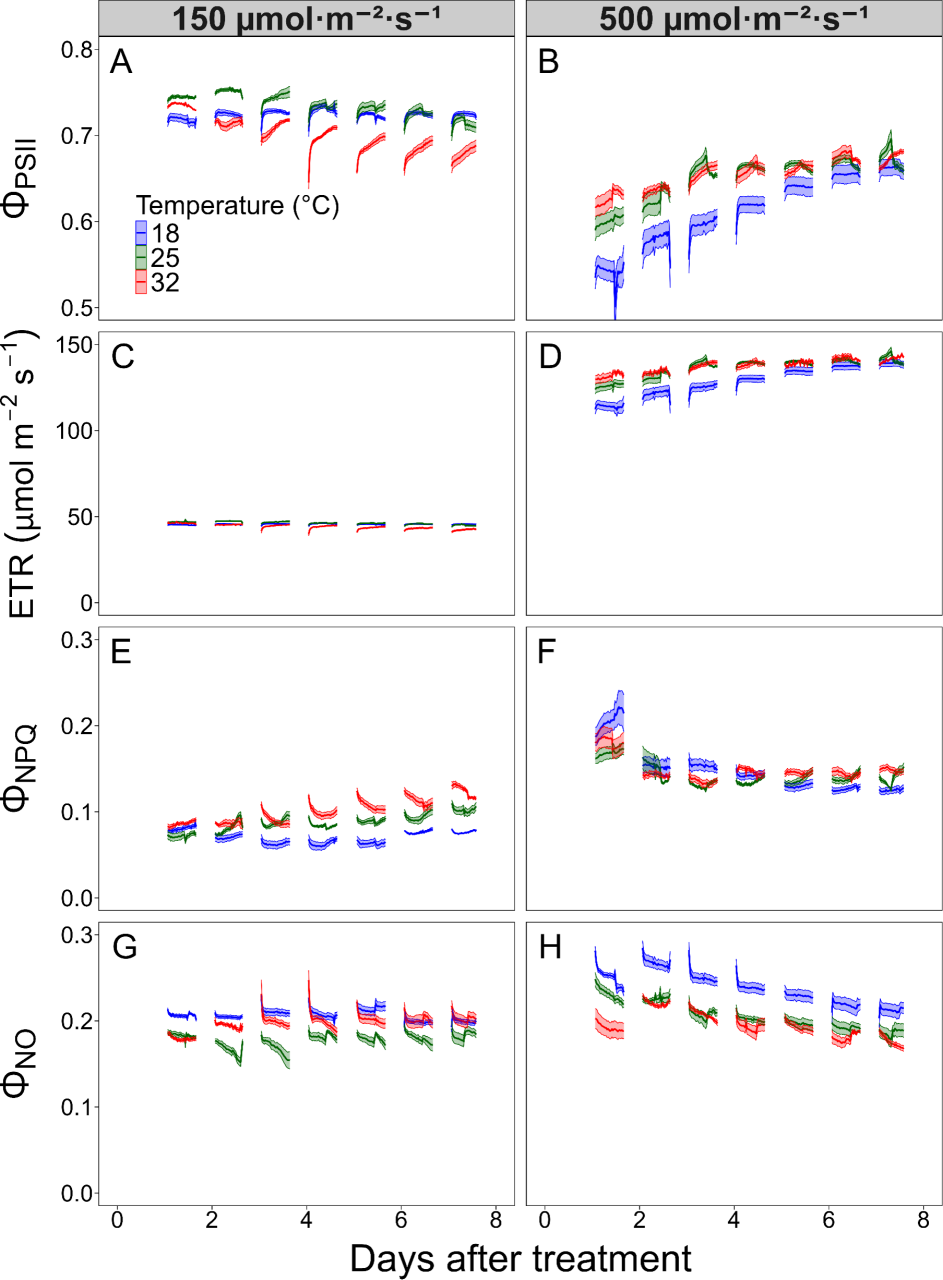
Chlorophyll fluorescence parameters were measured every 30 minutes during a 16-hour photoperiod in lettuce (*Lactuca sativa* ‘Green Towers’) grown under two photosynthetic photon flux densities (PPFD) (150 and 500 μmol·m^-2^·s^-1^) and three temperature conditions (18, 25, and 32 °C) for seven days. Data were collected for the operating photosystem II (PSII) efficiency (Φ_PSII_) **(A, B)**, electron transport rate (ETR) **(C, D)**, quantum yield of non-photochemical quenching (Φ_NPQ_) **(E, F)**, and quantum yield of non-regulated energy dissipation (Φ_NO_) **(G, H)**. Solid lines indicate mean values, and shaded areas indicate standard errors (n = 4). Some standard error areas are not visible due to their minimal size.

A real-time CF monitoring system (**Figure 1**) was used to collect high-temporal-resolution data on photochemical activity every 30 minutes during the photoperiod and every hour at night. This setup enabled continuous monitoring of CF over a 7-day period, capturing both daily and within-day changes under different temperature and light conditions (**Figure 2**). Φ_PSII_ gradually increased during the daytime within each day, particularly under 32°C with low light and under high light (500 μmol·m^-2^·s^-1^ PPFD) across all temperatures, suggesting short-term photochemical adjustments to heat and light stress (**Figures 2A, B**). These diurnal changes were associated with a diurnal decrease in Φ_NPQ_ and Φ_NO_, reflecting a shift in energy distribution from non-photochemical to photochemical processes (**Figures 2E–H**). Notably, a distinct zigzag pattern in Φ_PSII_ was observed under 32°C and 150 μmol·m^−2^·s^−1^ PPFD, characterized by a daytime increase followed by an abrupt decrease the next morning, leading to an overall decline from DAT 2 to 7. In contrast, plants under 500 μmol·m^−2^·s^−1^ PPFD showed a more continuous increase in Φ_PSII_, with minimal discrepancies between days.

### 3.2 Photosynthetic efficiency and energy dissipation

**Figure 3:**
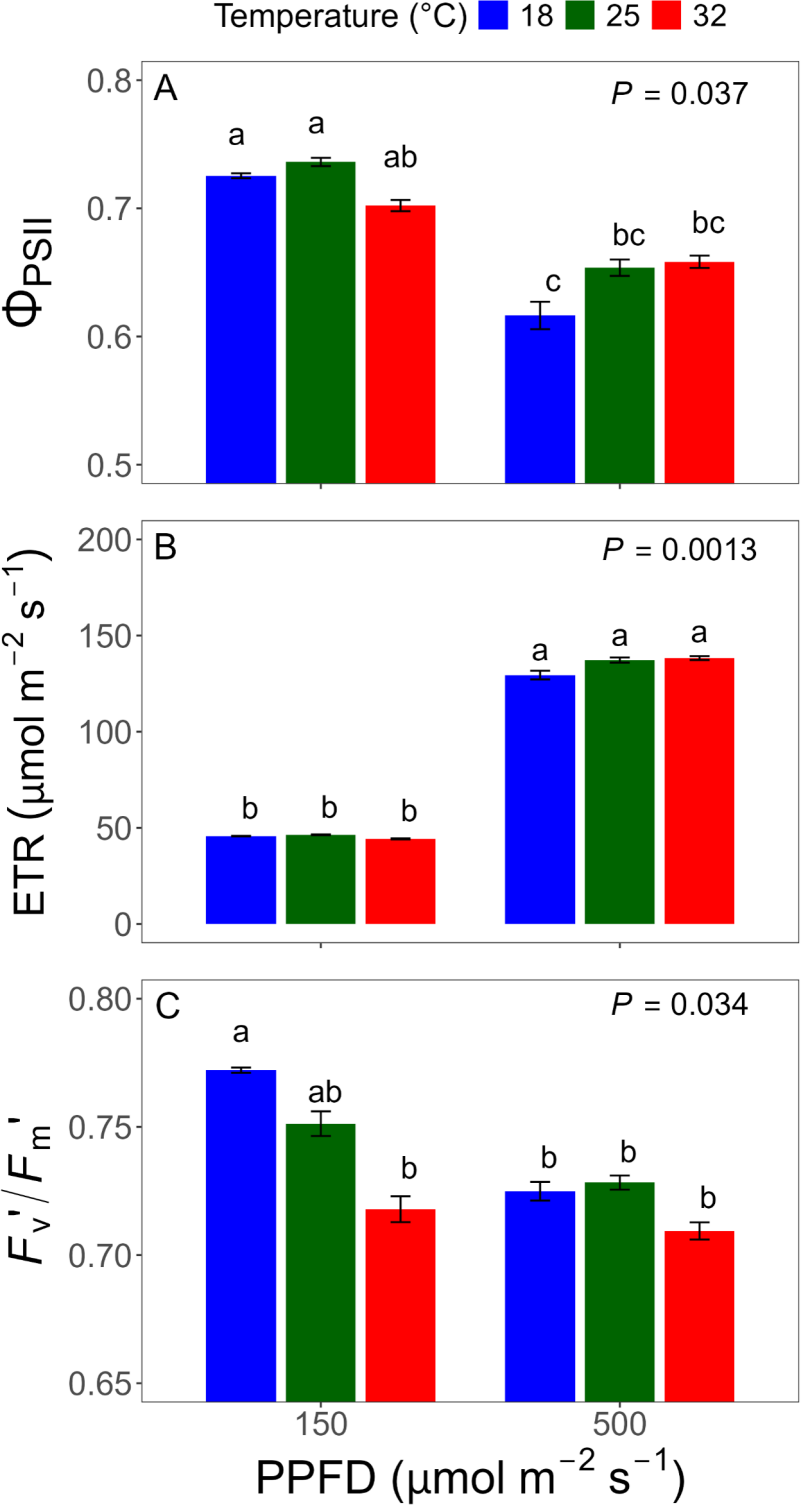
Chlorophyll fluorescence parameters for temperature × photosynthetic photon flux density (PPFD) interactions were statistically significant. Data represent means of operating photosystem II (PSII) efficiency (Φ_PSII_) **(A)**, electron transport rate (ETR) **(B)**, and light-adapted maximum quantum efficiency (*F*_v_′/*F*_m_′) **(C)**, averaged across days after treatment. Different letters indicate significant differences (*P* < 0.05) based on Tukey’s Honestly Significant Difference (HSD) test. Error bars represent standard errors (n = 4).

Overall, Φ_PSII_ was lower under 500 μmol·m^-2^·s^-1^ than under 150 μmol·m^-2^·s^-1^, and there was no temperature effect alone (**Table 1**), but the temperature effect varied depending on PPFD (**Figures 2A, B**). At 150 μmol·m^-2^·s^-1^, Φ_PSII_ was lowest at 32°C, whereas at 500 μmol·m^-2^·s^-1^, it was lowest at 18°C. Across all conditions, 25°C consistently showed the highest Φ_PSII_, indicating that specific combinations of light intensity and temperature could be suboptimal for maintaining photochemical efficiency in lettuce. Although Φ_PSII_ tended to decrease at extreme temperatures, these differences were not statistically significant when averaged across time (**Figure 3A**).

**Table 1:** Effects of temperature, photosynthetic photon flux density (PPFD), and days after treatment (DAT) on daily averaged chlorophyll fluorescence parameters; operating photosystem II (PSII) efficiency (Φ_PSII_), electron transport rate (ETR), quantum yield of non-photochemical quenching (Φ_NPQ_), quantum yield of non-regulated energy dissipation (Φ_NO_), light-adapted maximum quantum efficiency (*F*_v_′/*F*_m_′), 1-hour dark-adapted maximum quantum efficiency (*F*_v_/*F*_m_ 1h), 8-hour dark-adapted maximum quantum efficiency (*F*_v_/*F*_m_ 8h), and photochemical quenching coefficient (qL).

Treatments		Φ_PSII_	ETR	Φ_NPQ_	Φ_NO_	*F*_v_′/*F*_m_′	*F*_v_/*F*_m_ 1h	*F*_v_/*F*_m_ 8h	qL
Temperature (T)	18 °C	0.670 ± 0.011 a	87.5 ± 2.3 a	0.109 ± 0.006	0.221 ± 0.006 a	0.748 ± 0.003 a	0.753 ± 0.006 b	0.820 ± 0.003 ab	0.707 ± 0.022 b
	25 °C	0.695 ± 0.003 a	91.8 ± 0.8 a	0.114 ± 0.003	0.191 ± 0.005 b	0.740 ± 0.003 a	0.776 ± 0.003 a	0.831 ± 0.001 a	0.806 ± 0.031 ab
	32 °C	0.680 ± 0.005 a	91.3 ± 0.8 a	0.124 ± 0.004	0.195 ± 0.002 b	0.714 ± 0.003 b	0.757 ± 0.003 ab	0.813 ± 0.002 b	0.869 ± 0.009 a
PPFD (P)	150 μmol·m^-2^·s^-1^	0.722 ± 0.002 a	45.5 ± 0.1 b	0.086 ± 0.003 b	0.193 ± 0.003 b	0.747 ± 0.002 a	0.778 ± 0.003 a	0.820 ± 0.003 a	0.874 ± 0.011 a
	500 μmol·m^-2^·s^-1^	0.642 ± 0.004 b	134.9 ± 0.9 a	0.146 ± 0.003 a	0.212 ± 0.002 a	0.721 ± 0.001 b	0.746 ± 0.002 b	0.823 ± 0.001 a	0.714 ± 0.010 b
*P*-values									
	*T*	*0.025*	*0.019*	*0.61*	*0.028*	*0.014*	*< 0.001*	*0.0078*	*0.016*
	*P*	*< 0.001*	*< 0.001*	*< 0.001*	*< 0.001*	*< 0.001*	*< 0.001*	*0.0054*	*< 0.001*
	*DAT*	*0.49*	*0.50*	*0.78*	*0.33*	*0.94*	*0.76*	*0.013*	*0.26*
	*T × P*	*0.037*	*0.0013*	*0.29*	*0.30*	*0.034*	*0.17*	*0.070*	*0.75*
	*T × DAT*	*< 0.001*	*< 0.001*	*0.0037*	*0.13*	*< 0.001*	*< 0.001*	*0.010*	*0.97*
	*P × DAT*	*< 0.001*	*< 0.001*	*< 0.001*	*0.0076*	*< 0.001*	*< 0.001*	*< 0.001*	*< 0.001*
	*T × P × DAT*	*0.88*	*0.079*	*0.74*	*0.83*	*0.67*	*0.58*	*0.95*	*0.60*

Values are means ± standard error (n = 4), and different letters within a column from the same factor indicate significant differences at α = 0.05, according to Tukey’s Honestly Significant Difference (HSD) test.

**Figure 4:**
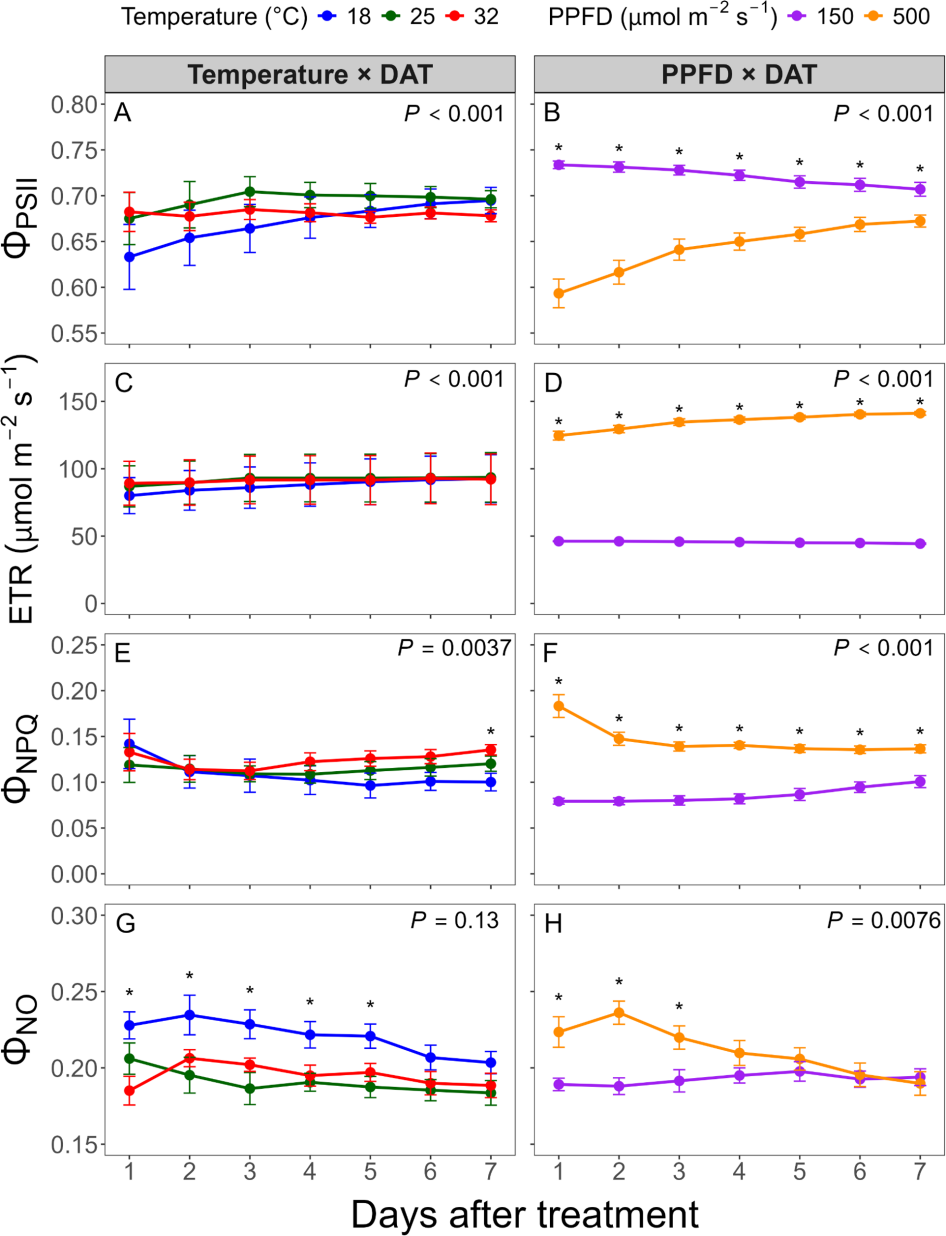
Daily mean values of chlorophyll fluorescence parameters measured over seven days after treatment (DAT). Parameters include operating photosystem II (PSII) efficiency (Φ_PSII_) **(A, B)**, electron transport rate (ETR) **(C, D)**, quantum yield of non-photochemical quenching (Φ_NPQ_) **(E, F)**, and quantum yield of non-regulated energy dissipation (Φ_NO_) **(G, H)**. Left panels **(A, C, E, G)** show temperature × DAT interactions averaged across photosynthetic photon flux density (PPFD) levels; right panels **(B, D, F, H)** show PPFD × DAT interactions averaged across temperature levels. Asterisks indicate significant differences between treatments at each time point (*P* < 0.05). Error bars represent standard errors (n = 4).

Daily means of CF values were calculated to assess the effects of temperature, light intensity, and DAT, as the 30-minute interval data showed high autocorrelation and were difficult to analyze directly (**Figure 4**). Φ_PSII_ was also affected by DAT, with trends depending on both temperature and PPFD. At 25 and 32°C, Φ_PSII_ remained relatively constant over time, while values at 18°C increased by approximately 10%, suggesting a recovery response (**Figure 4A**). Under 500 μmol·m^-2^·s^-1^ PPFD, Φ_PSII_ gradually increased by about 14% over the 7-day period, while under 150 μmol·m^-2^·s^-1^, it decreased slightly by less than 4% (**Figure 4B**). Initially, Φ_PSII_ under 150 μmol·m^-2^·s^-1^ was 24% higher than under 500 μmol·m^-2^·s^-1^, but the difference narrowed to only 5% by the end of the experiment. This suggests that plants showed the most pronounced acclimation over time in photosynthetic efficiency under high light and low temperature.

ETR was approximately three times higher under 500 μmol·m^-2^·s^-1^ compared to 150 μmol·m^-2^·s^-1^, with average values of approximately 134 and 45 μmol·m^-2^·s^-1^, respectively (**Table 1**). Compared to Φ_PSII_, the effects of temperature and DAT on ETR were relatively minor (**Figures 3B**, **4C**), but followed the same overall pattern of Φ_PSII_, as ETR is calculated from Φ_PSII_, incident PPFD, and fixed coefficients.

Φ_NPQ_ and Φ_NO_ represent the proportion of absorbed light dissipated as heat or lost through unregulated pathways rather than being used for photochemistry. Both parameters were significantly higher under 500 μmol·m^-2^·s^-1^ compared to 150 μmol·m^-2^·s^-1^, indicating that higher light intensity increased non-photochemical energy dissipation, which was associated with reduced photosynthetic efficiency (**Figures 4F, H**).

Over time, Φ_NPQ_ and Φ_NO_ decreased under 500 μmol·m^-2^·s^-1^, corresponding with a gradual increase in Φ_PSII_, suggesting acclimation to high light. In contrast, under 150 μmol·m^-2^·s^-1^, Φ_NPQ_ and Φ_NO_ remained stable, resulting in stable Φ_PSII_. Temporal trends differed between the two parameters. Under 500 μmol·m^-2^·s^-1^, Φ_NPQ_ exhibited a significant decrease during the first day and remained stable thereafter (**Figure 4F**), while Φ_NO_ gradually decreased throughout the experiment (**Figure 4H**). As a result, by the end of the 7-day period, there was no significant difference in Φ_NO_ between the two light conditions. Temperature effects on Φ_NPQ_ over time were not clearly distinct, but at the end of the experiment, Φ_NPQ_ was highest at 32°C, followed by 25 and 18°C (**Figure 4E**). In contrast, Φ_NO_ remained highest at 18°C throughout most of the experiment period, though differences among temperatures were no longer significant by DAT 6 and 7.

### 3.3 Temporal changes in maximum quantum efficiency

**Figure 5:**
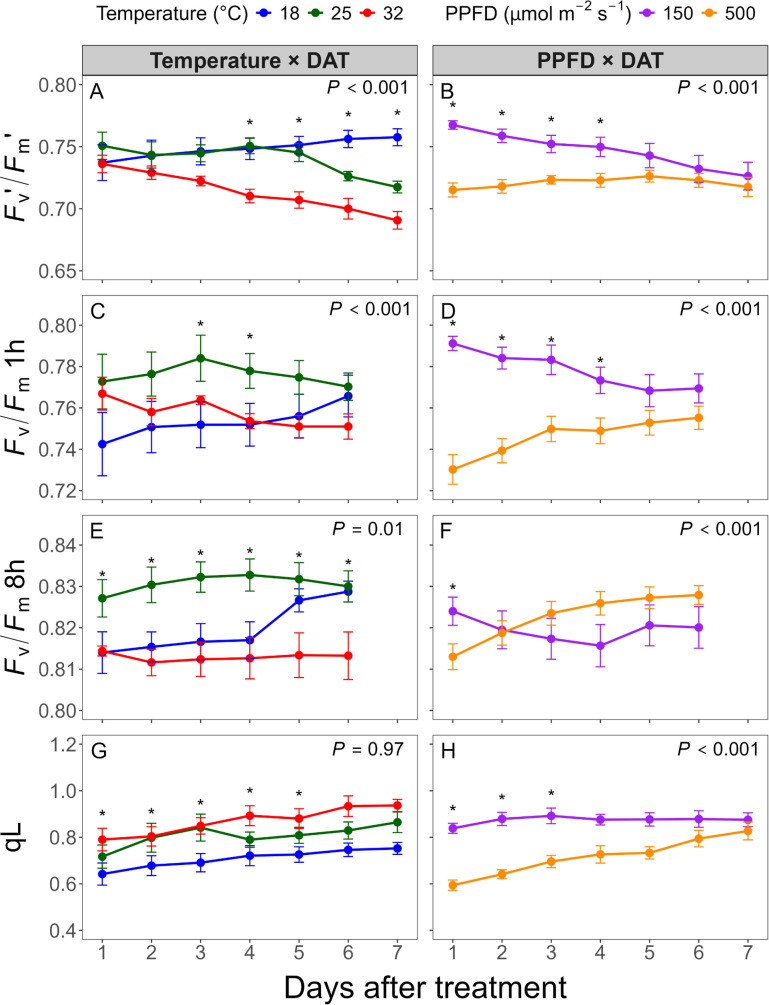
Daily mean values of chlorophyll fluorescence parameters measured over seven days after treatment (DAT). Parameters include light-adapted maximum quantum efficiency (*F*_v_′/*F*_m_′) **(A, B)**, 1-hour dark-adapted *F*_v_/*F*_m_ (*F*_v_/*F*_m_ 1h) **(C, D)**, 8-hour dark-adapted *F*_v_/*F*_m_ (*F*_v_/*F*_m_ 8h) **(E, F)**, and photochemical quenching coefficient (qL) **(G, H)**. Left panels **(A, C, E, G)** show temperature × DAT interactions averaged across photosynthetic photon flux density (PPFD) levels; right panels **(B, D, F, H)** show PPFD × DAT interactions averaged across temperature levels. Asterisks indicate significant differences between treatments at each time point (*P* < 0.05). Error bars represent standard errors (n = 4).

Daily mean values of *F*_v_′/*F*_m_′, an estimate of maximum quantum efficiency at a given light intensity if all PSII centers were open, showed a different trend compared to Φ_PSII_. *F*_v_′/*F*_m_′ was higher under 150 than 500 μmol·m^-2^·s^-1^, but gradually decreased over time, whereas it remained relatively stable under 500 μmol·m^-2^·s^-1^ (**Figure 5B**). Temperature effects were also observed: *F*_v_′/*F*_m_′ was highest at 18°C, followed by 25 and 32°C under 150 μmol·m^-2^·s^-1^ (**Figure 3C**), with increasing values at 18 °C and decreasing trends at 25 and 32°C (**Figure 5A**).

The average *F*_v_/*F*_m_ across all treatments and DAT was 0.762 after 1 hour of dark adaptation, but stabilized at 0.821 from 2 through 8 hours after dark adaptation, indicating that full recovery was reached within the initial two hours. Therefore, *F*_v_/*F*_m_ 1h and *F*_v_/*F*_m_ 8h were selected to assess transient and sustained effects of environmental stress. Under 150 μmol·m^-2^·s^-1^, *F*_v_/*F*_m_ decreased over time, while it increased under 500 μmol·m^-2^·s^-1^ (**Figures 5D, F**). *F*_v_/*F*_m_ 1h was consistently higher under 150 μmol·m^-2^·s^-1^, but *F*_v_/*F*_m_ 8h under 500 μmol·m^-2^·s^-1^ recovered fully, approaching 0.83, the commonly accepted optimal value. *F*_v_/*F*_m_ 1h and *F*_v_/*F*_m_ 8h were highest at 25°C and lower under suboptimal temperatures (**Figures 5C, E**). However, *F*_v_/*F*_m_ 8h at 18°C recovered to approximately 0.83 by DAT 6 and 7, while values at 32°C remained low around 0.813, suggesting more persistent stress at higher temperatures despite a sufficient dark adaptation period.

qL, the proportion of open PSII reaction centers, also showed clear temperature and light effects. qL was highest at 32°C, followed by 25 and 18°C, and these temperature-dependent differences remained nearly constant throughout the experiment (**Figure 5G**). Under 150 μmol·m^-2^·s^-1^, qL remained stable, whereas it gradually increased under 500 μmol·m^-2^·s^-1^ (**Figure 5H**). Consequently, the large initial difference between light intensities diminished by DAT 3.

### 3.4 Leaf gas exchange parameters

**Figure 6:**
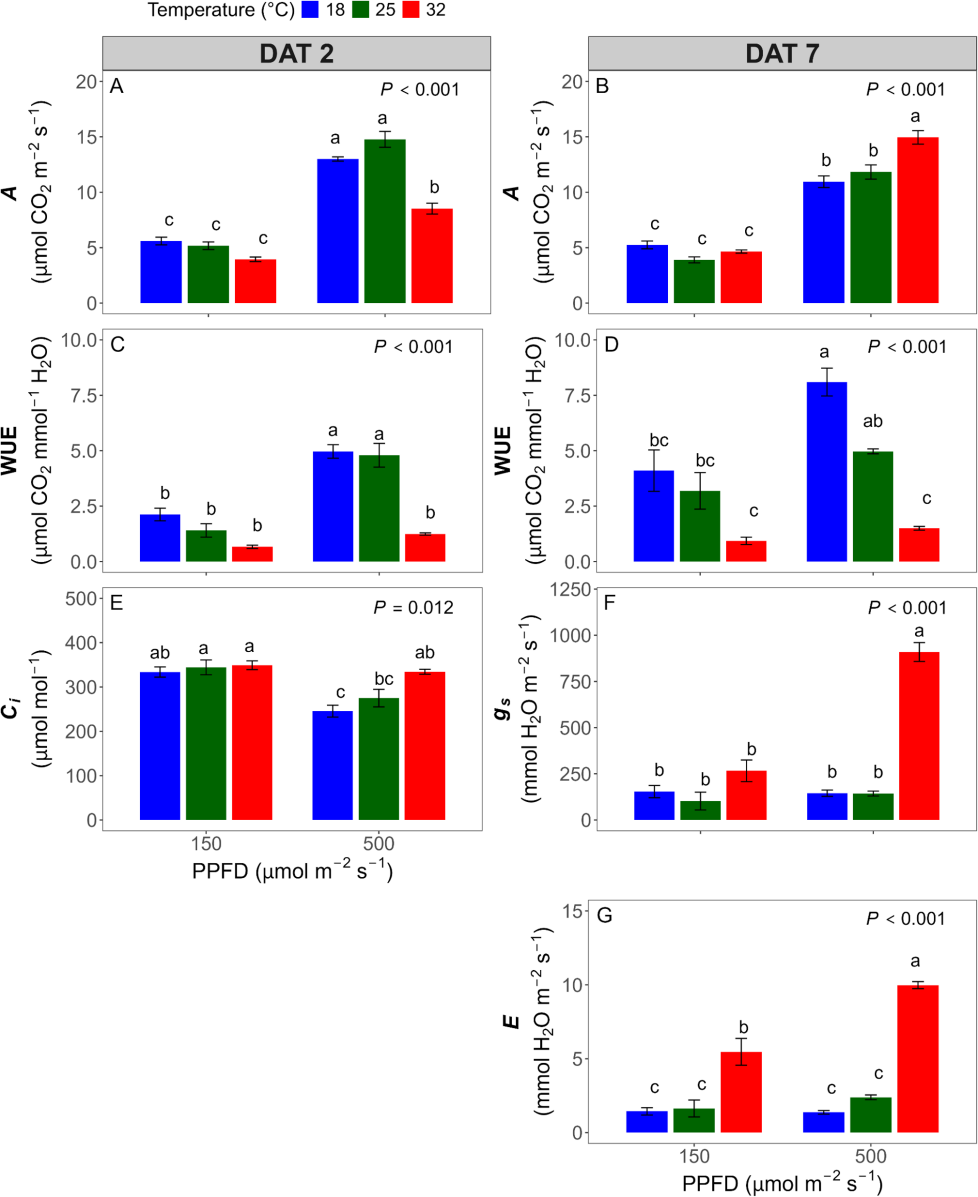
Leaf gas exchange parameters measured at two days **(A, C, E)** and seven days **(B, D, F, G)** after treatment. Shown are parameters for which temperature × photosynthetic photon flux density (PPFD) interactions were statistically significant: photosynthetic rate (*A*) **(A, B)**, water use efficiency (WUE) **(C, D)**, intercellular carbon dioxide (CO_2_) concentration (*C*_i_) **(E)**, stomatal conductance (*g*_s_) **(F)**, and transpiration rate (*E*) **(G)**. Different letters denote significant differences based on Tukey’s Honestly Significant Difference (HSD) test. (*P* < 0.05). Error bars represent standard error (n = 4).

leaf gas exchange measurements were conducted to examine whether the effects of temperature and light intensity observed in CF were also reflected in CO_2_ assimilation and stomatal responses. Measurements were taken on DAT 2 and 7 to capture both early stress responses and potential acclimation (**Figure 6**).The *A* was consistently higher at 500 than under 150 μmol·m^-2^·s^-1^ at both time points, regardless of temperature (**Figures 6A, B**). However, temperature effects varied depending on light intensity and time. Under 150 μmol·m^-2^·s^-1^, temperature differences were minimal. In contrast, under 500 μmol·m^-2^·s^-1^, *A* was lowest at 32°C on DAT 2 but became highest on DAT 7, indicating recovery or acclimation at high temperature.

*g*_s_ increased with temperature on DAT 2, with no significant effect of PPFD (**Table 2**). By DAT 7, under 32°C and 500 μmol·m^-2^·s^-1^, the *g*_s_ increased by 900 mmol·H_2_O·m^-2^·s^-1^ and was 5.6 times higher than in other treatment combinations (**Figure 6F**). These results indicate strong stomatal adjustment under combined high temperature and high light over time. *E* followed a similar pattern to *g*_s_, increasing with temperature across both DATs and PPFD levels. In particular, at DAT 7 under 500 μmol·m^-2^·s^-1^, the *E* was 5.3 times higher at 32°C compared to the lower temperatures (**Figure 6G**). On both DAT 2 and 7, *C*_i_ was lower under 500 than under 150 μmol·m^-2^·s^-1^, and 32 °C had the highest *C*_i_ across temperature treatments (**Figure 6E**). The overall mean *C*_i_ decreased from 314 to 231 µmol·mol^-1^ from DAT 2 to DAT 7. WUE declined with increasing temperature on both dates, with consistently higher values under 500 μmol·m^-2^·s^-1^. The temperature effect on WUE was more pronounced under high light, while differences under 150 μmol·m^-2^·s^-1^ were not statistically significant (**Figures 6C, D**) (*P* > 0.05).

**Table 2:** Effects of temperature and photosynthetic photon flux density (PPFD) on leaf photosynthesis parameters; Photosynthetic rate (*A*), stomatal conductance (*g*_s_), transpiration rate (*E*), intercellular carbon dioxide (CO_2_) concentration (*C*_i_), and water use efficiency (WUE). Measurements were taken on days after treatment (DAT) 2 and 7, and the data were analyzed separately for each day.

DAT	Treatments		*A* (μmol·CO_2_·m^-2^·s^-1^)	*g*_s_ (mol·H_2_O·m^-2^·s^-1^)	*E* (mmol·H_2_O·m^-2^·s^-1^)	*C*_i_ (μmol·mol^-1^)	WUE (μmol·CO_2_· mmol^-1^·H_2_O)
DAT 2	Temperature (T)	18 °C	9.30 ± 0.22 a	145 ± 6 b	2.72 ± 0.20 b	290 ± 6 b	3.55 ± 0.16 a
		25 °C	9.98 ± 0.42 a	301 ± 40 ab	3.64 ± 0.20 b	310 ± 15 ab	3.10 ± 0.12 a
		32 °C	6.24 ± 0.21 b	393 ± 58 a	6.54 ± 0.48 a	342 ± 7 a	0.95 ± 0.06 b
	PPFD (P)	150 μmol·m^-2^·s^-1^	4.91 ± 0.16 b	280 ± 41	4.34 ± 0.24	342 ± 11 a	1.40 ± 0.06 b
		500 μmol·m^-2^·s^-1^	12.10 ± 0.40 a	279 ± 44	4.26 ± 0.27	285 ± 5 b	3.67 ± 0.14 a
	*P*-values						
		*T*	*< 0.001*	*< 0.001*	*< 0.001*	*< 0.001*	*< 0.001*
		*P*	*< 0.001*	*0.99*	*0.84*	*< 0.001*	*< 0.001*
		*T × P*	*< 0.001*	*0.17*	*0.24*	*0.012*	*< 0.001*
DAT 7	Temperature (T)	18 °C	8.10 ± 0.40 b	150 ± 21 b	1.41 ± 0.16 b	223 ± 12 b	6.10 ± 0.73 a
		25 °C	7.86 ± 0.24 b	123 ± 25 b	2.01 ± 0.27 b	194 ± 13 b	4.08 ± 0.40 a
		32 °C	9.80 ± 0.29 a	588 ± 9 a	7.72 ± 0.38 a	275 ± 5 a	1.22 ± 0.08 b
	PPFD (P)	150 μmol·m^-2^·s^-1^	4.60 ± 0.19 b	174 ± 18 b	2.85 ± 0.26 b	256 ± 9 a	2.74 ± 0.25 b
		500 μmol·m^-2^·s^-1^	12.58 ± 0.16 a	399 ± 12 a	4.58 ± 0.11 a	205 ± 4 b	4.86 ± 0.18 a
	*P*-values						
		*T*	*< 0.001*	*< 0.001*	*< 0.001*	*< 0.001*	*< 0.001*
		*P*	*< 0.001*	*< 0.001*	*< 0.001*	*< 0.001*	*< 0.001*
		*T × P*	*< 0.001*	*< 0.001*	*< 0.001*	*0.47*	*< 0.001*

Values are means ± standard error (n = 4). For each DAT, different letters within a column from the same factor indicate significant differences at α = 0.05, according to Tukey’s Honestly Significant Difference (HSD) test.

**Figure 7:**
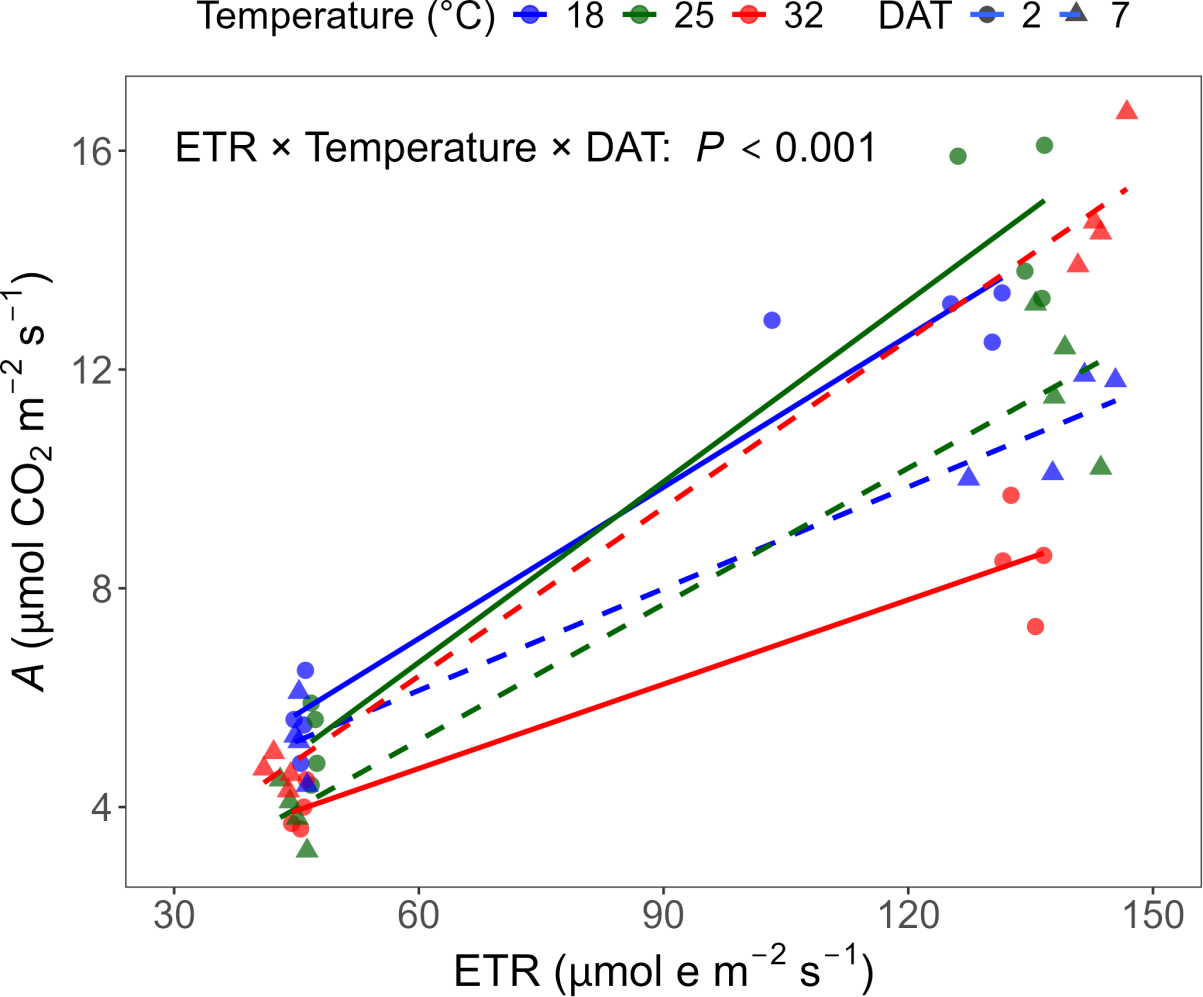
Relationship between photosynthetic rate (*A*) and electron transport rate (ETR) measured on days after treatment (DAT) 2 and 7. Each data point represents an individual replicate (n = 4) from each treatment combination. A linear regression model was used to assess the effect of ETR on *A* as influenced by temperature and DAT. Solid lines with circles indicate DAT 2, while dashed lines with triangles indicate DAT 7.

The relationship between ETR and *A* was significantly affected by temperature (*P* < 0.001) and the interaction between temperature and DAT (*P* < 0.001), but not by DAT alone (*P* = 0.31). Here, the slope of the ETR-*A* relationship reflects the efficiency with which electrons derived from the photosynthetic electron transport chain are utilized for carbon assimilation (**Figure 7**). At DAT 2, the slope was lowest at 32°C, indicating reduced CO_2_ assimilation per unit of electron transport at high temperature. By DAT 7, the slope at 32°C increased and exceeded those at 18 and 25°C, while the slopes at 18 and 25°C decreased over time. These results suggest that the coupling between electron transport and CO_2_ fixation was initially suppressed at high temperature but improved with acclimation.

**Figure 8:**
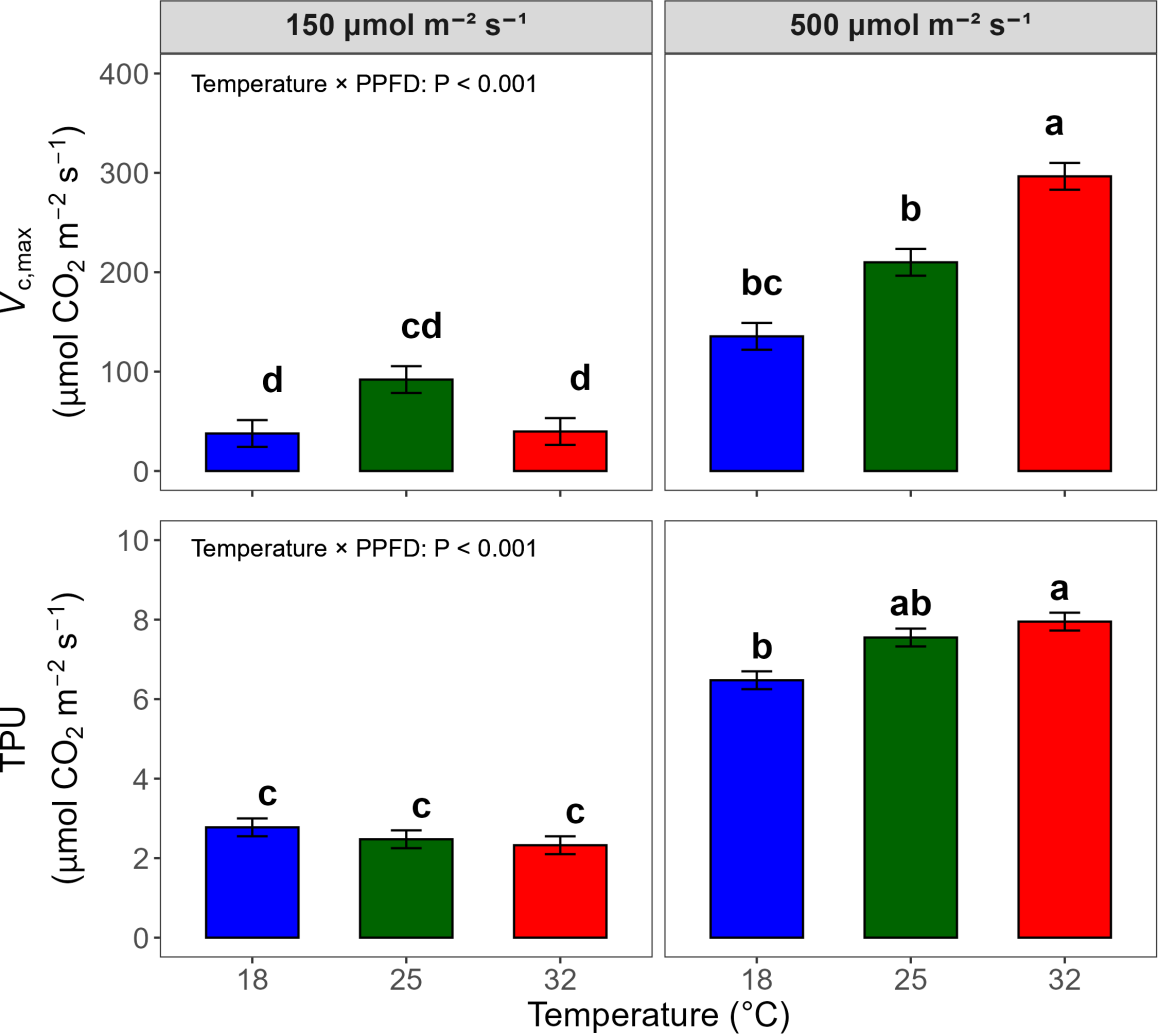
Bar graphs of maximum rate of carboxylation (*V*_c,max_) and triose phosphate utilization rate (TPU) measured on DAT 7, analyzed by two-way analysis of variance (ANOVA) to assess temperature × photosynthetic photon flux density (PPFD) interactions. Bars represent means (n = 4), and different letters indicate significant differences among treatment combinations based on Tukey’s Honestly Significant Difference (HSD) test (*P* < 0.05). Error bars represent standard errors.

*A*/*C*_i_ response curves were used to illustrate photosynthetic responses across treatments (**Supplementary Figure 1**). Overall, 500 μmol·m^-2^·s^-1^ resulted in greater *A* values across the *C*_i_ range. Temperature had no significant main effect on *A*, but a significant interaction between temperature and light intensity was observed (*P* = 0.019), with reduced *A* at 32°C under low light and at 18°C under high light. *V*_c,max_, *J*, and TPU were estimated from the *A*/*C*_i_ curves (**Table 3**). *V*_c,max_ was 3.8 times higher under 500 than 150 µmol m^-2^ s^-1^, but the temperature effect varied depending on light intensity (**Figure 8**). Under 500 µmol m^-2^ s^-1^, *V*_c,max_ increased with temperature and reached its highest value at 32°C. However, under 150 µmol m^-2^ s^-1^, *V*_c,max_ remained low across all temperatures *J* was strongly correlated with ETR (R^2^ = 0.92) and primarily influenced by light intensity, showing higher *J* under higher PPFD without significant temperature effects (**Table 3**). TPU also increased 2.9 times in response to high light intensity (*P* < 0.001), and a significant interaction was observed. TPU increased with temperature only under 500 μmol·m^-2^·s^-1^ (*P* < 0.001).

**Table 3:** Effects of temperature and photosynthetic photon flux density (PPFD) on maximum rate of carboxylation (*V*_c,max_), maximum electron transport rate (*J*), and triose phosphate utilization rate (TPU), measured on DAT 7.

Treatments		*V*_c,max_ (μmol·CO_2_·m^-2^·s^-1^)	*J* (μmol·m^-2^·s^-1^)	TPU (μmol·CO_2_·m^-2^·s^-1^)
Temperature (T)	18 °C	86.6 ± 5.3 b	103.8 ± 5.1	4.63 ± 0.21
	25 °C	151.0 ± 13.4 a	103.0 ± 3.1	5.01 ± 0.12
	32 °C	168.1 ± 8.4 a	92.9 ± 4.2	5.14 ± 0.18
PPFD (P)	150 μmol·m^-2^·s^-1^	56.5 ± 5.1 b	51.3 ± 3.2 b	2.53 ± 0.03 b
	500 μmol·m^-2^·s^-1^	214.0 ± 10.9 a	148.5 ± 5.0 a	7.33 ± 0.20 a
*P*-values				
	*T*	*< 0.001*	*0.18*	*0.057*
	*P*	*< 0.001*	*< 0.001*	*< 0.001*
	*T × P*	*< 0.001*	*0.22*	*< 0.001*

Values are means ± standard error (n = 4), and different letters within a column from the same factor indicate significant differences at α = 0.05, according to Tukey’s Honestly Significant Difference (HSD) test.

### 3.5 Growth responses

**Figure 9:**
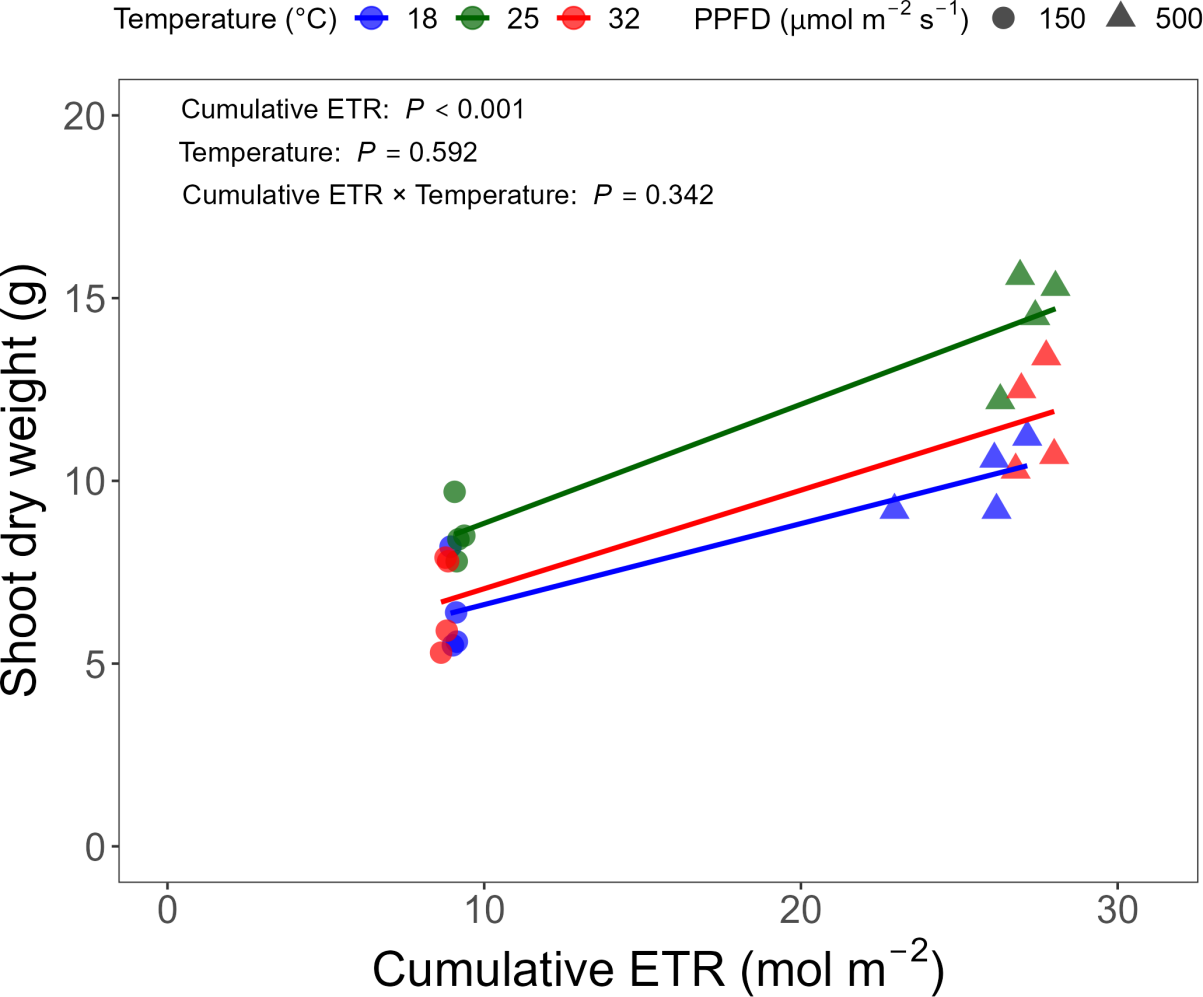
Relationship between cumulative electron transport rate (ETR) and shoot dry weight. Each point represents an individual replicate (n = 4) for each treatment combination. A linear regression model was used to assess the effect of cumulative ETR on shoot dry weight and its interaction with temperature. Circles and triangles denote photosynthetic photon flux density (PPFD) of 150 and 500 μmol·m^-2^·s^-1^, respectively.

Shoot biomass was highest at 25 °C and under 500 μmol·m^-2^·s^-1^, with shoot dry weight reduced by 28% and 20% at 18°C and 32°C compared to 25°C (**Table 4**). Shoot dry weight increased significantly with increasing cumulative ETR (*P* < 0.001; **Figure 9**). After accounting for cumulative ETR, temperature did not have a significant additional effect on shoot dry weight (*P* = 0.592), and the relationship between cumulative ETR and shoot dry weight did not differ among temperature treatments (*P* = 0.342). Although shoot fresh weight decreased by only 18% under 150 μmol·m^-2^·s^-1^, shoot dry weight decreased by 40%. This discrepancy may be explained by higher shoot water content under low light. Likewise, shoot water content was also highest at the favorable temperature, 25°C. Chlorophyll content was slightly higher under 500 μmol·m^-2^·s^-1^, with no significant effect of temperature. Although temperature × light intensity interactions were significant for both chlorophyll content and shoot water content, the magnitudes were minimal (**Supplementary Figure 2**).

**Table 4:** Effects of temperature and photosynthetic photon flux density (PPFD) on shoot fresh weight, shoot dry weight, chlorophyll content, and shoot water content, measured at harvest on days after treatment (DAT) 7.

Treatments		Shoot fresh weight (g)	Shoot dry weight (g)	Shoot water content (%)	Chlorophyll content (CCI)
Temperature (T)	18 °C	112.2 ± 8.4 b	8.24 ± 0.56 b	92.7 ± 0.3 b	32.2 ± 0.4
	25 °C	196.2 ± 9.0 a	11.50 ± 0.40 a	94.2 ± 0.2 a	33.2 ± 2.5
	32 °C	134.9 ± 6.3 b	9.23 ± 0.52 b	93.2 ± 0.2 b	30.2 ± 0.7
PPFD (P)	150 μmol·m^-2^·s^-1^	133.5 ± 6.2 b	7.25 ± 0.32 b	94.5 ± 0.1 a	29.3 ± 1.1 b
	500 μmol·m^-2^·s^-1^	162.0 ± 4.9 a	12.06 ± 0.34 a	92.3 ± 0.1 b	34.4 ± 0.8 a
*P*-values					
	*T*	*< 0.001*	*< 0.001*	*< 0.001*	*0.38*
	*P*	*0.0014*	*< 0.001*	*< 0.001*	*< 0.001*
	*T × P*	*0.10*	*0.13*	*0.031*	*0.012*

Values are means ± standard error (n = 4), and different letters within a column from the same factor indicate significant differences at α = 0.05, according to Tukey’s Honestly Significant Difference (HSD) test.

## 4 Discussion

### 4.1 Light intensity effect on photochemistry

Although photosynthesis increases with light intensity, the photosynthetic efficiency often declines under high light due to excess excitation energy that cannot be used for electron transport and carbon fixation. This energy is instead dissipated via non-photochemical pathways, such as regulated heat dissipation (Φ_NPQ_) and unregulated energy loss (Φ_NO_). As shown in previous studies (Kramer et al., 2004), Φ_NPQ_ increases proportionally with light intensity, whereas Φ_NO_ remains relatively unchanged, suggesting that Φ_NPQ_ plays a larger role in reducing photosynthetic efficiency under high light. It is consistent with our observation of elevated Φ_NPQ_ but relatively similar Φ_NO_ under high light conditions compared to low light (**Table 1**).

Over time, both Φ_NPQ_ and Φ_NO_ declined under high light, while Φ_PSII_ increased, indicating dynamic acclimation (**Figure 4**). NPQ decreased rapidly and stabilized after DAT 2, which may be attributed to various physiological responses, such as fast adjustment of the NPQ component, energy-dependent quenching (qE), through lumen acidification, PSII subunit S (PsbS) activation, and the xanthophyll cycle (Murchie and Niyogi, 2011; Zuo, 2025). NPQ is a protective mechanism that safely dissipates excess light as heat, preventing photodamage caused by unregulated energy loss. Hence, moderately high NPQ under high light is not necessarily detrimental, although it reduces photosynthetic efficiency.

In contrast, Φ_NO_ continuously declined throughout the week, suggesting that plants systematically adjusted the photosynthetic machinery and improved light use efficiency through processes associated with changes in light harvesting and electron transport capacity, such as reduced light-harvesting complex II (LHCII) antenna size, chloroplast repositioning, and modulation of electron transport enzymes (Murchie and Niyogi, 2011). The sustained increase in Φ_PSII_ after DAT 2 appeared more closely associated with the decline in Φ_NO_ than with changes in NPQ. This highlights that obtaining Φ_NPQ_ and Φ_NO_ data separately provides insights into the different types of mechanisms driving improvements in photosynthetic efficiency.

qL, which reflects both open PSII reaction centers and the redox state of the plastoquinone (PQ) pool, increased over time under high light, paralleling improvements in Φ_PSII_ (**Figure 5H**). This recovery may result from enhanced PQ biosynthesis, which replenishes the oxidized PQ pool and facilitates electron transport, as previously reported by Yang et al. (2022).

*F*_v_′/*F*_m_′ represents the theoretical maximum efficiency of PSII in the light-adapted state and declines further when severe photoinhibition or electron transport limitations occur after NPQ is saturated (Baker et al., 2007). Its stability under high light (**Figure 5B**) in this study suggests PSII capacity was preserved, likely due to effective NPQ regulation.

Determining accurate *F*_v_/*F*_m_ requires a sufficient period of dark adaptation, however, 15–30 minutes is commonly used. Residual qI from prolonged stress, the slowly reversible component of NPQ, can persist for several hours (Tietz et al., 2017). Willits and Peet (2001) emphasized that complete dark adaptation is necessary for the full oxidation of electron carriers and dissipation of the proton gradient. In our study, *F*_v_/*F*_m_ continued to increase beyond 1 h and stabilized after 2 h, indicating that shorter dark periods may make it difficult to distinguish between transient and persistent stress.

Under high light, initial photoinhibition (**Figures 5D, F**) likely resulted from pre-acclimation to 250 μmol·m^-2^·s^-1^ in the walk-in growth chamber. Plants that had been previously grown under low light were more susceptible to photoinhibition even under moderate intensities (Gjindali and Johnson, 2023). As acclimation progressed, photochemical efficiency improved through the repair mechanism, resulting in ~ 0.83 of *F*_v_/*F*_m_ from DAT 4. Under low light, *F*_v_/*F*_m_ gradually declined but did not fall below 0.82, suggesting no severe photodamage. Instead, this pattern may reflect a physiological shift toward maximizing light capture (e.g., increasing antenna size), potentially at the expense of electron transport and carbon fixation capacity (Gjindali and Johnson, 2023).

### 4.2 Temperature and light combination effect on photochemistry

Temperature influenced photochemistry less dynamically than light intensity, yet clear stress responses were observed under suboptimal thermal conditions. Low temperature initially suppressed Φ_PSII_, mainly due to increased Φ_NO_ rather than Φ_NPQ_, indicating excess energy loss through non-regulated pathways and photoinhibition. Over time, however, Φ_PSII_ increased substantially as Φ_NO_ decreased, suggesting photosynthetic acclimation. According to previous studies, such recovery under cold stress has been associated with enhanced repair and metabolic adjustments, including reduced antenna size, increased expression of the cytochrome b_6_f complex and Rubisco, and higher ATP synthase activity, which have been reported to improve electron transport and carbon fixation capacity (Bascuñán-Godoy et al., 2012).

Cold stress impairs enzyme activity in the Calvin-Benson cycle and reduces thylakoid membrane fluidity, limiting electron transport and PSII function (Fracheboud and Leipner, 2003). Low qL values under low temperature conditions in this study may indicate an over-reduction of the PQ pool and a potential accumulation of ROS. However, during acclimation, increased qL with decreased Φ_NO_ suggests a shift toward more efficient electron transport and reduced risk of photodamage (Bascuñán-Godoy et al., 2012; Mattila et al., 2020). The gradual recovery of *F*_v_/*F*_m_ under low temperature stress reflects repair of PSII components such as D1 and cytochrome b_6_f (Bascuñán-Godoy et al., 2012; DeEll and Toivonen, 2003).

In contrast, high temperature resulted in the highest qL values but declining *F*_v_′/*F*_m_′ and *F*_v_/*F*_m_ (**Figure 5**), suggesting heat-induced inhibition of electron transport despite maintained reaction center openness (Mathur et al., 2014). Heat stress affects PQ oxidation, leading to singlet oxygen accumulation, plastoquinol degradation, and inhibition of Calvin-Benson cycle activity by Rubisco deactivation, thereby disrupting nicotinamide adenine dinucleotide phosphate (NADPH) consumption and redox balance (Rath et al., 2022; Sharkey and Zhang, 2010). These effects can reduce PSII efficiency, with a quadratic decrease in *F*_v_/*F*_m_ as temperature increases (Fracheboud and Leipner, 2003; Willits and Peet, 2001).

Notably, combinations of suboptimal light and temperature amplify stress responses. Under low temperatures with high light, Φ_PSII_ was the lowest and Φ_NO_ was the highest, indicating photoinhibition due to excess light energy and limited photochemical capacity (**Figures 2B, H**). Conversely, high temperature combined with low light also impaired photochemistry (**Figure 2A**). Under this condition, *F*_v_′/*F*_m_′ was particularly reduced, potentially due to limited light harvesting capacity paired with sustained thermal inhibition of photosynthesis and reduced chlorophyll content (Gjindali and Johnson, 2023). While high light can drive photoprotection, low light combined with high temperature reduces chlorophyll content and Calvin cycle enzyme activity, leading to further suppression of photochemical activity (Zhou et al., 2022).

Overall, while high temperatures had limited effects on Φ_PSII_ in this study (**Figure 4A**), the observed decline in *F*_v_/*F*_m_ 8h (~0.81) suggests moderate but persistent stress. Relatively low heat tolerance of lettuce may explain the limited recovery observed, as its acclimation capacity under heat stress is less dynamic compared to cold stress or high light acclimation (Hermida-Carrera et al., 2016; Lu et al., 2017).

### 4.3 Linking CF data to carbon assimilation and growth

Gas exchange partially tracked CF trends but diverged under temperature extremes. Carbon assimilation followed a parabolic temperature response, with suppression at both low and high temperatures (Yamori et al., 2014). At 18 °C, Φ_PSII_ was reduced in the early stage (**Figure 4A**), but *A* remained similar to 25 °C (**Figure 6A**), and *g*_s_ and *C*_i_ showed no strong limitation (**Table 2**), suggesting that photochemical inhibition at low temperature did not constrain CO_2_ fixation. Previous studies have reported that reduced photochemistry under cold conditions can be compensated by lower dark respiration, reduced photorespiration, and sustained chlorophyll levels (Yamori et al., 2014; Zhou et al., 2022). At low temperature, an increased CO_2_/O_2_ solubility ratio favors Rubisco carboxylation over oxygenation, which is generally associated with reduced photorespiration and helps sustain carbon assimilation efficiency under cold conditions (Sage and Kubien, 2007). From DAT 2 to 7, *A* slightly declined at both 18 and 25 °C (**Table 2**) despite stable ETR (**Figures 4C, D**), indicating possible feedback inhibition due to sink limitation or TPU bottlenecks (Adler et al., 2025). This imbalance between electron transport and carbon fixation likely contributed to the gradual decline in the ETR/*A* slope (**Figure 7**).

In contrast, high temperature induced transient inhibition of carbon assimilation followed by strong recovery. At 32 °C, *A* was initially suppressed significantly (**Figure 6A**) despite only slight reductions in Φ_PSII_ (**Figure 4A**), indicating a temporary decoupling between photochemistry and carbon assimilation. Although *g*_s_, *E*, and *C*_i_ were elevated at 32 °C on DAT 2, *A* remained suppressed, indicating that carbon assimilation was initially limited by non-stomatal factors such as enhanced photorespiration or reduced Rubisco activity rather than CO_2_ diffusion (Sharkey and Zhang, 2010; Yamori et al., 2014). As acclimation progressed, *A* increased substantially by DAT 7 particularly under the high temperature and high light combination (**Figure 6B**), coinciding with pronounced increases in *g*_s_ and E (**Figures 6F, G**), which in turn increased the slope of the *A* vs. ETR relationship (**Figure 7**). Compared to *g*_s_ and *E*, *C*_i_ remained slightly higher under the high temperature but was lower under high light intensity, suggesting that increased CO_2_ diffusion through stomatal opening was largely balanced by enhanced carbon assimilation rates. This adaptation was also supported by higher *V*_c,max_ and TPU at elevated temperatures with high light combination at DAT 7 (**Figure 8**), indicating metabolic acclimation of carbon assimilation (Farquhar et al., 1980; Urban et al., 2017). Together, these stomatal and biochemical adjustments led to a disproportionate increase in carbon assimilation relative to photochemical efficiency, resulting in a divergence between *A* and Φ_PSII_ observed under high temperature conditions.

Biomass and shoot water content revealed a decoupling from photosynthetic rate. Although Φ_PSII_ at 18 °C recovered to levels comparable to 25 °C, and *A* at 32 °C exceeded that at 25 °C by DAT 7, shoot biomass remained highest at 25 °C (**Table 4**). This indicates that improved photochemical efficiency or CO_2_ assimilation did not necessarily translate into biomass accumulation. This may reflect limitations imposed by downstream processes such as respiratory carbon losses or carbon allocation dynamics (Seydel et al., 2022), although these mechanisms were not directly assessed in this study. Shoot water content was also reduced under thermal extremes (**Table 4**), likely due to transpirational loss under heat and impaired water uptake or cell expansion under cold (Yu et al., 2025). High light further decreased water content and increased chlorophyll concentration, consistent with physiological and structural acclimation observed in this study (Zha et al., 2019; Zhou et al., 2022).

Taken together, these results indicate that instantaneous CF parameters and gas exchange responses do not necessarily reflect final growth outcomes. However, cumulative ETR, integrated over the experimental period, emerged as a useful predictor of biomass accumulation independent of temperature (**Figure 9**). This result indicates that integrating photochemical performance across time retains useful information relevant to biomass accumulation. Previous studies have suggested that the predictive value of CF parameters for crop yield improves when temporal dynamics are incorporated (Moriyuki and Fukuda, 2016). In particular, Moriyuki and Fukuda highlighted the importance of developing a high-resolution, time-course CF measurement system to evaluate the potential of CF-based indices for growth prediction, rather than relying on single time-point observations.

Importantly, the cumulative ETR–biomass relationship identified here was derived from short-term (7-day) experiments under controlled conditions. While these results demonstrate the potential of temporally integrated CF metrics to link photochemical performance with biomass accumulation, further validation over longer growth periods and across different crop species and cultivars is required to assess the broader applicability of this approach.

### 4.4 High-temporal resolution CF monitoring system for real-time growing condition control

CF offers a comprehensive and reliable method for assessing photochemical efficiency and plant stress responses. Unlike gas exchange measurements, CF provides detailed information on the function of PSII and electron transport, including parameters such as Φ_PSII_, Φ_NPQ_, Φ_NO_, qL, and *F*_v_′/*F*_m_′, which help characterize how different stress conditions affect photosynthetic capacity (Kalaji et al., 2016). These indicators reveal whether stress is transient or sustained, photoinhibitory or protective, which gas exchange measurements alone cannot distinguish. A decline in *F*_v_/*F*_m_ is a widely accepted marker of photoinhibition under temperature and light stress (Maxwell and Johnson, 2000). In this study, *A* recovered over time under the high temperature and high light combination due to increased *g*_s_, but *F*_v_/*F*_m_ remained low, indicating sustained photoinhibitory damage that would have been overlooked using CO_2_ exchange alone (Willits and Peet, 2001).

Conventional methods for determining *F*_v_/*F*_m_ require a long period of dark adaptation, which would interrupt ongoing light treatments if measurements are conducted during the photoperiod (Willits and Peet, 2001), while measurements on detached leaves may not reflect real-time physiological responses (Yu and Chen, 2023). Instead, parameters such as *F*_v_′/*F*_m_′ and qL can provide insights into PSII photochemical efficiency and electron transport limitations (Baker et al., 2007), but they require accurate measurement of *F*_o_′, which can only be obtained when all PSII reaction centers are open and is therefore challenging under ambient light conditions (Kramer et al., 2004). In this study, far-red LED lighting was integrated into the CF monitoring system in the growth chamber (**Figure 1**), enabling rapid and automated estimation of *F*_o_′ and consequently *F*_v_′/*F*_m_′ and qL without long dark adaptation or interruption of daytime conditions (Maxwell and Johnson, 2000; Tietz et al., 2017).

Real-time, high-frequency CF monitoring enabled early detection of plant stress and subtle changes over time in photosynthetic performance. Unlike conventional gas exchange measurements, which are typically conducted at daily or less frequent intervals, CF can be collected non-destructively at much higher temporal resolution (Maxwell and Johnson, 2000; Moustaka and Moustakas, 2023). In this study, CF parameters were measured every 30 minutes, enabling the visualization of diurnal patterns and stress dynamics in real-time. This level of detail is critical for detecting rapid photochemical responses and interpreting acclimation processes. The system’s flexibility, enabled by serial communication between the chlorophyll fluorometer and the datalogger, allows customized measurement regimes based on experimental objectives (Nam et al., 2025; van Iersel et al., 2016).

Real-time CF data can support adaptive control of growing conditions in CEA systems. CF monitoring systems have the potential to optimize energy use, maximize productivity, and protect plants from environmental stress by responding to photochemical indicators such as Φ_PSII_ and *F*_v_′/*F*_m_′ (Ahlman et al., 2017; Baker and Rosenqvist, 2004). In previous studies, CF-based biofeedback systems were used to adjust LED lighting intensity to maintain target ETR or Φ_PSII_ as plant responses changed, reducing energy use while sustaining photosynthetic performance (Nam et al., 2025; van Iersel et al., 2016). For example, based on our results, high light combined with low temperature induced photoinhibition, which could be mitigated by temporarily dimming LED lights. As plants acclimate and recover their photosynthetic efficiency, light intensity can be gradually restored or even increased to promote growth. In vertical farms, continuous lighting with a 24-h photoperiod and spectra with a high blue fraction or ultraviolet (UV) LED lighting are being actively investigated for their effects on crop productivity and quality. Under these conditions, real-time CF monitoring combined with biofeedback light control can be used to diagnose light stress and track acclimation processes, allowing dynamic adjustment of light intensity and spectrum based on photochemical responses. Such plant-driven lighting strategies may help prevent severe photoinhibition while maintaining photosynthetic performance. This approach can also be extended to a comprehensive CEA environmental control system using CF parameters to regulate not only lighting but also temperature, ventilation, and irrigation, applicable in both vertical farming systems and greenhouses (Nam et al., 2025; van Iersel et al., 2016).

## 5 Conclusion

This study applied high-temporal resolution CF monitoring to evaluate photosynthetic responses to varying light and temperature conditions. High light and low temperature stress initially reduced PSII efficiency, but gradual acclimation over one week was observed, indicated by decreases in Φ_NO_ and increases in *F*_v_/*F*_m_, rather than by changes in NPQ. In contrast, high temperature with high light increased CO_2_ assimilation markedly over time due to enhanced stomatal conductance, yet *F*_v_/*F*_m_ remained suppressed, suggesting sustained photoinhibition. The high-frequency CF monitoring system, operating every 30 minutes, enabled the real-time detection of subtle changes and diurnal trends in photochemical responses, capturing stress responses and acclimation patterns that would not have been evident from daily measurements or gas exchange data alone. The integration of light-adapted parameters requiring far-red illumination, such as qL and *F*_v_′/*F*_m_′, allowed for a more comprehensive interpretation of photochemical limitations and electron transport efficiency. Although CF parameters did not always align with downstream carbon assimilation or biomass accumulation, they provided valuable insights into photochemical bottlenecks and stress status. These findings highlight the potential of CF monitoring to support real-time environmental decision-making in CEA systems, for example, by identifying photochemical stress thresholds that could inform adaptive adjustments in supplemental light intensity or thermal management.

## Data Availability

The raw data supporting the conclusions of this article will be made available by the authors, without undue reservation.
