# Supplementary material for: Real-time chlorophyll fluorescence monitoring reveals dynamic acclimation of lettuce to temperature and light stress in controlled environments
Source: Front Plant Sci. 2026 Feb 10;17:1733839. doi: 10.3389/fpls.2026.1733839 (PMC12929122; doi:10.3389/fpls.2026.1733839)
Supplement: Supplementary file 1 [file Supplementaryfile1.docx]

Supplementary Material

# Supplementary Figures


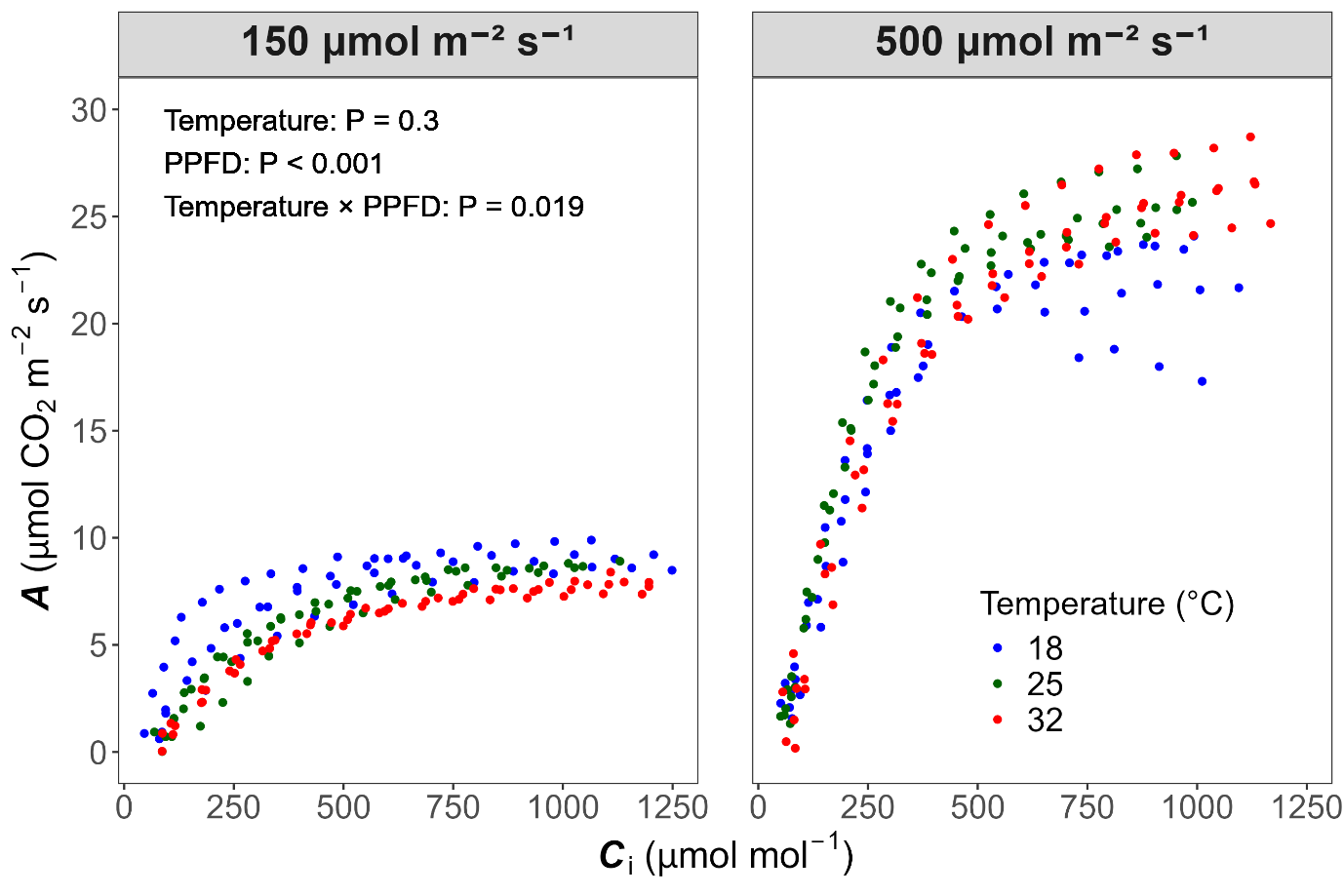


**Supplementary Figure 1.** Photosynthetic carbon dioxide (CO_2_) response (*A*/*C*_i_) curves measured on days after treatment (DAT) 7. Measurements were conducted using a rapid CO_2_ ramping technique. Statistical analysis was performed using a two-way analysis of variance (ANOVA) to assess the effects of temperature and photosynthetic photon flux density (PPFD) on photosynthetic rate. Data points represent individual measurements from all replicates (n = 4) without averaging.


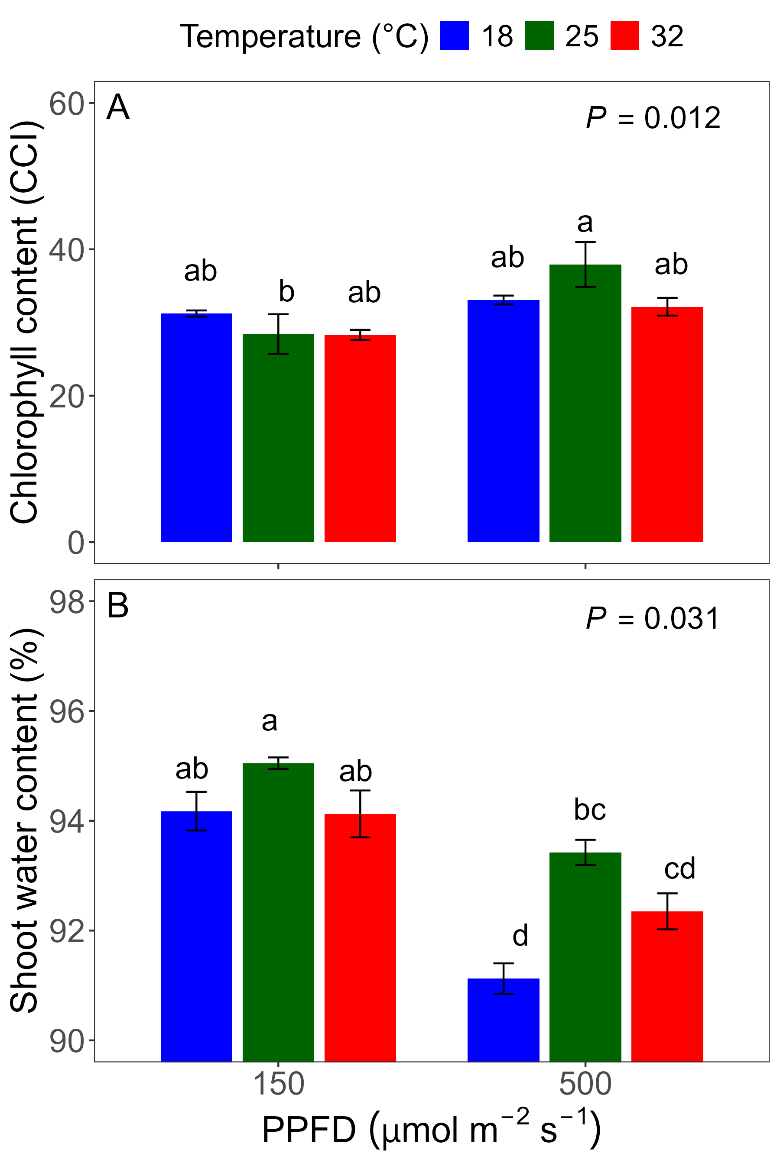


**Supplementary Figure 2.** Chlorophyll content and shoot water content measured on days after treatment (DAT) 7, analyzed by two-way analysis of variance (ANOVA) to assess temperature × photosynthetic photon flux density (PPFD) interactions. Bars represent means ± standard error (n = 4), and different letters indicate significant differences among treatment combinations based on Tukey’s Honestly Significant Difference (HSD) test (P < 0.05). Error bars represent standard error.
